# Patient-reported outcomes for patients with metastatic castration-resistant prostate cancer receiving docetaxel and Atrasentan versus docetaxel and placebo in a randomized phase III clinical trial (SWOG S0421)

**DOI:** 10.1186/s41687-018-0054-5

**Published:** 2018-06-13

**Authors:** Joseph M. Unger, Katherine Griffin, Gary W. Donaldson, Karen M. Baranowski, Margorie J. Good, Eunicia Reburiano, Maha Hussain, Paul J. Monk, Peter J. Van Veldhuizen, Michael A. Carducci, Celestia S. Higano, Primo N. Lara, Catherine M. Tangen, David I. Quinn, James L. Wade, III, Nicholas J. Vogelzang, Ian M. Thompson, Jr, Carol M. Moinpour

**Affiliations:** 1 SWOG Statistics and Data Management Center, Fred Hutchinson Cancer Research Center, Seattle, WA USA; 2 University of Utah, Salt Lake City, UT USA; 3 Karmanos Cancer Center, Farmington Hills, MI USA; 4 National Cancer Institute, Washington, DC USA; 5 ICON PLCC, Philadelphia, PA USA; 6 Robert H. Lurie Comprehensive Cancer Center of Northwestern University, Chicago, IL USA; 7 The Ohio State University James Cancer Hospital, Columbus, OH USA; 8 Sarah Cannon Cancer Center, Kansas City, KS USA; 9 Johns Hopkins University School of Medicine, Baltimore, MD USA; 10 Pacific Cancer Research Consortium NCORP, Seattle Cancer Care Alliance, University of Washington, Seattle, WA USA; 11 University of California at Davis, Sacramento, CA USA; 12 University of Southern California Norris Comprehensive Cancer Center, Los Angeles, CA USA; 13 Heartland NCORP, Decatur, IL USA; 14 US Oncology Research Comprehensive Cancer Centers, Las Vegas, NV USA; 15 CHRISTUS Santa Rosa Hospital Medical Center, San Antonio, TX USA; 16 Fred Hutchinson Cancer Research Center, Seattle, WA USA; 17 Fred Hutchinson Cancer Research Center, M3-C102/P.O. Box 19024, 1100 Fairview Avenue North, Seattle, WA 98109-1024 USA

**Keywords:** Patient-reported outcomes, Health-related quality of life (HRQL), Pain, Functional status, Cancer clinical trial, Metastatic castration-resistant prostate Cancer (mCRPC)

## Abstract

**Background:**

SWOG S0421 was a large randomized trial comparing docetaxel/prednisone plus placebo (DPP) to docetaxel/prednisone plus atrasentan over 12 cycles for patients with metastatic castration-resistant prostate cancer (mCRPC). The current report presents the PRO results for this trial, an important secondary endpoint.

**Methods:**

The trial specified two primary PRO endpoints. Palliation of worst pain was based on the Brief Pain Inventory (BPI), where a 2 point difference is defined as clinically meaningful. Improvement of functional status was based on the Functional Assessment of Cancer Therapy – Prostate Cancer Trial Outcome Index (FACT-P TOI); a 5-point difference has been defined as clinically meaningful. We compared rates by arm using chi-square tests. Longitudinal analyses using linear mixed models addressed changes by arm over time.

**Results:**

Four-hundred eighty-nine patients on each arm were evaluable for PRO endpoint data. There were no differences by arm in clinically meaningful pain palliation (41.7% for DPP vs. 44.0% for DPA, *p* = .70) or functional status (24.2% for DPP vs. 28.7% for DPA, *p* = .13). Longitudinal comparisons indicated no differences over time by arm for BPI Worst Pain scores (0.13 points, *p* = .23). Patients on the DPA arm had improved functional status of 1.78 points on average, a statistically significant (*p* = .02) but not clinically meaningful difference.

**Conclusions:**

The SWOG S0421 PRO data showed little evidence of clinically meaningful differences by arm in either pain palliation or functional status.

**Electronic supplementary material:**

The online version of this article (10.1186/s41687-018-0054-5) contains supplementary material, which is available to authorized users.

## Background

Patients with metastatic castration-resistant prostate cancer (mCRPC) are no longer sensitive to a continued regimen of androgen deprivation treatment (ADT). This condition is defined based on confirmed objective progression and/or increases in prostate specific antigen (PSA) in the presence of castrate ranges of testosterone; bone metastases are often present. Bone is the most common site for metastasis in prostate cancer disease and is associated with significant morbidity, the most common of which is pain [1]. There are a number of FDA- approved new agents for the treatment of this disease, including docetaxel, which was first approved in 2004.

The inclusion of Patient-Reported Outcomes (PROs) in studies of prostate cancer are vital for informing trial interpretations, as they provide key insights into patients’ symptoms and treatment response [2]. Men with prostate cancer face issues that differ substantially depending on stage of disease; early stage patients receiving local treatment to the prostate experience urinary, sexual, and bowel problems, while men in the advanced care setting experience systemic bone pain and symptomatic deterioration [3]. A recent review by Nussbaum et al. concluded that PROs improved the determination of treatment impact in the mCRPC medical setting by providing a more comprehensive evaluation of new treatments [4]. Newer agents used to treat prostate cancer tend to generate fewer toxicities with chemotherapy and corresponding better or stable health-related quality of life (HRQL) [5]. In mCRPC trials, PROs can be especially useful for informing the assessment of the risk/benefit tradeoff from the patient’s perspective for treatment regimens with modest effects on overall survival [6, 7].

This report describes the PROs for SWOG S0421, a study designed to examine whether the addition of atrasentan to docetaxel would increase progression-free survival and overall survival compared to docetaxel and a matching placebo for atrasentan. SWOG S0421 clinical outcomes were previously reported [8]. SWOG S0421 was based on SWOG S9916, an earlier trial of treatment for androgen-independent prostate cancer, that compared docetaxel/estramustine versus mitoxantrone/prednisone [9] and found no differences by arm in either pain palliation or global HRQL [10].

## Methods

### Patient population and study design

SWOG S0421 was activated in August, 2006 and closed in May, 2010. In total, 1038 patients with mCRPC were randomized to docetaxel (75 mg/m^2^ every 21 days, intravenously) with atrasentan

(10 mg/day, orally) (DPA) or placebo (DPP) for up to 12 cycles administered every 3 weeks of 36 weeks [8]. Continued treatment with either atrasentan or placebo could occur for a maximum of 52 weeks for patients who did not progress after 36 weeks. The addition of atrasentan to docetaxel/prednisone did not confer better overall or progression-free survival; statistically significant differences in toxicity were not observed by treatment arm [8]. The study protocol was approved by Institutional Review Boards at the participating institutions enrolling cancer patients on the trial. All patients who participated in the trial and who could complete PRO forms signed detailed consent forms for the PRO component of the trial. Given the importance of reporting standards for methodological transparency, this report follows recommendations described in the CONSORT PRO Extension [11].

### Protocol-specified PRO outcomes

Major secondary objectives of the SWOG S0421 trial included PRO measures to monitor palliation of bone pain and improvement in functional status. Multiple PRO measurements were administered: a Symptom Questionnaire (comprised of the Brief Pain Inventory (BPI) [12], and the SF-36 Vitality scale [13]); the Functional Assessment of Cancer Therapy – Prostate (FACT-P) [14, 15]; and a Pain Medication Log [16].

### Earlier and newer research supporting use of the protocol-specified PRO outcomes

The PRO outcome measures to assess pain, functional status, and vitality are consistent with the currently recommended measures suggested for studies of treatment for prostate cancer described by the various working groups. A National Cancer Institute (NCI) Symptom Management and Health-related Quality of Life Steering Committee Working Group on the inclusion of PRO measures in prostate cancer clinical trials recommended assessing four domains in prostate cancer clinical trials (physical and mental well-being, fatigue, and pain) [3]. The European Expert Consensus Panel for the Management of Metastatic CRPC recommended that PROs (pain and HRQL) were appropriate as important secondary outcomes in Phase III trials [17]. The Prostate Cancer Clinical Trials Working Group 3 recommended the use of PRO assessment of adverse events [18]. The Working Group also emphasized the importance of measuring physical function; the FACT-General Physical and Functional Well-being scales address this PRO domain [13]. The recommendations also suggested that levels of baseline pain (or other symptoms of interest) must be clinically meaningful and that change must be based on well-specified response criteria [6]. Finally, the main PROs selected for this trial include those areas of change that Eton et al. (2010) documented for patients being treated for metastatic hormone-refractory prostate cancer [19].

#### Pre-specified primary PRO measures

##### Pain response

Pain was measured with the BPI Short Form, the psychometric properties of which have been previously documented [11]. Among its items, the BPI asks about a patient’s worst pain in the past 24 h. The BPI Worst Pain item is measured on a “0” to “10” response scale, with higher scores reflecting more pain or more interference with functioning. A two-point reduction in the Worst Pain rating has been documented as clinically significant [9, 20–22].

##### Functional status - FACT-P trial outcome index (FACT-P TOI)

Functional status was assessed by the FACT-P. This questionnaire addresses four general PRO domains (physical, functional, emotional, and social well-being subscales) as well as symptom concerns associated with prostate cancer and its treatment [13, 14]. Higher scores reflect better functioning. The “primary” functional status outcome was the FACT-P TOI score. The TOI score, calculated with three FACT-P subscales (functional and physical well-being and prostate symptom concerns) is sensitive to change in clinical status (e.g., performance status and PSA) and its components reflect the key areas of importance to prostate cancer patients as noted above [15]. We used a five-point difference [14] to compare observed differences in TOI scores for the two treatment arms; this difference corresponds to a 0.4 to 0.5 effect size, which represents a medium effect and a clinically meaningful difference [14]. A more recent publication indicated that change scores of five to nine points are more appropriate for determining clinically meaningful change for the FACT-P TOI [23].

#### Pre-specified secondary PRO measures

##### Vitality

The total score for the four-item Medical Outcomes Study Short Form-36 (SF-36) Energy/Vitality Scale ranges from “0” to “100”, with higher scores reflecting more vitality [12]. The SF-36 Energy/Vitality scale has acceptable psychometric properties and has been found to be a unidimensional construct addressing energy/vitality [12, 24].

##### General quality of life/health status

Two items from the EORTC QLQ-C30 were also included to address global or overall health and quality of life during the past week [25].

##### Analgesic use

Patient analgesic use was monitored using Pain Medication Logs. The Pain Medication Log was developed for use in SWOG S0421 and modified for ease of data entry [15]. Clinic staff were instructed to write the names of all pain medications the patient was taking on the Pain Medication Log: each pain medication was then categorized as a “0” (no pain medications), “1” (non-narcotic medication), “2” (weak opioid pain medication), and “3” (strong opioid pain medication). Medication type (pill, liquid, pump, and patch) and dose units were also transcribed. The patient recorded the number of pain medications taken in the 24 h prior to the clinic visit using the list of medications prepared by the clinic staff.

### Administration schedule

The Symptom Questionnaire was administered at baseline and at the end of each of the 12 cycles. The FACT-P was administered every three cycles (cycles 4 [week 10], 7 [week 19], 10 [week 28]), and after the end of treatment [beginning of week 37]. Additional follow-up was collected through week 52 but these data are not reported due to extensive missingness after 37 weeks (only *n* = 156 patients with QOL assessments at week 52 (16%)).

### Statistical methods and analysis

#### Statistical power

SWOG S0421 was expected to enroll 930 eligible patients. In total, 42% of patients were expected either to be asymptomatic at baseline (that is, having insufficient pain at baseline to feasibly detect pain reduction) or to drop out, resulting in 540 patients available for examinations of PRO endpoints. Data from Tannock et al. which included the control arm in this study (DPP), indicated that 35% of the patients in this arm achieved pain palliation [22]. Based on these data, the this study had 89% power to detect a 15% difference in the proportion of pain palliated patients (e.g., 35% versus 50%) using a two-sided test, based on the hypothesis was that worst pain reduction would be at least 15% more in the in the DPA arm compared to the DPP arm. For functional status, the five-point change noted above as clinically important for the FACT-P TOI score was used to evaluate the impact of treatment [14]. Five-hundred forty patients were sufficient for 92.5% power to detect an effect size of 0.42 (corresponding to 5 FACT-P TOI points) or 98.5% power to detect an effect size of 0.50 (corresponding to 6 FACT-P TOI points), based on the hypothesis that the FACT-P TOI score would be 6 points higher (more improved) than that for the DPP arm. Alpha = .025 tests were used to account for the two comparisons using Bonferonni adjustment.

#### Clinically meaningful change

To account for measurement error present in a single pain measure, the BPI Worst Pain baseline measure was defined as the average of the PreStudy and week 4 assessments for pain[26]. Successful pain palliation (a “responder”) was defined as a two-point reduction in pain maintained for two consecutive cycles through Week 37, with no evidence of an increase in analgesic use. A change of five points was specified in the protocol to indicate clinically significant change for the FACT-TOI from baseline to either week 28 or week 37. Chi-square analyses were conducted.

#### Longitudinal analyses

Longitudinal measures over time were described for the primary endpoint measures (BPI Worst Pain and FACT-P TOI), as well as secondary PRO measures including BPI pain interference, SF-36 energy/vitality, the EORTC global QOL score, the FACT-P Total Score, and the FACT-G emotional and social well-being subscales. For the primary endpoints, we examined longitudinal PRO measurements using linear mixed models using SAS PROC Mixed [27, 28]. We specified random effects for the intercept and slope and a compound symmetric correlation structure accounting for autocorrelation between serial observations within patients. For each outcome examined, models included intervention and assessment time as independent variables, as well as the baseline score. We also examined whether scores differed over time by arm using a treatment by time interaction (both linear and quadratic). Given more information due to serial measures over time, power for these analyses was higher than that specified for the primary endpoints, noted above. To account for potential informative missingness, we generated cohort plots to examine missing data patterns by arm over time; pattern mixture models (PMMs) were then conducted as a sensitivity analysis to identify whether estimates of study parameters substantially changed [29].

## Results

**Fig. 1:**
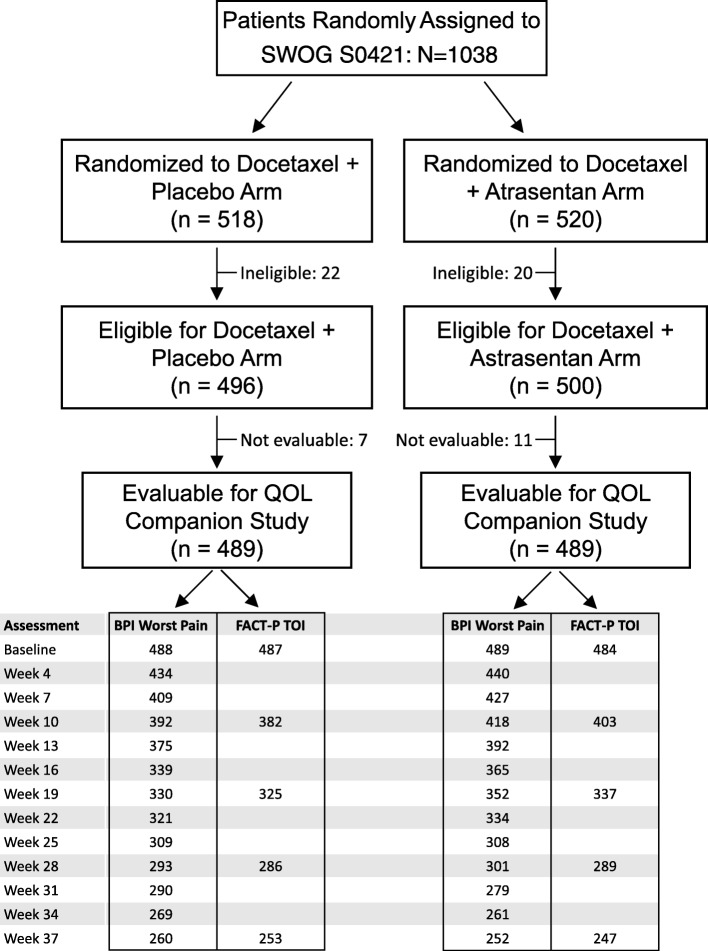
CONSORT diagram for patient-reported outcomes

S0421 was activated in August of 2006, with accrual closed in May of 2010. A total of 1038 patients were enrolled (518 on DPP and 520 on DPA), of whom 489 patients on each arm were evaluable for the QOL companion study (Fig. 1). Submission rates for the PRO primary endpoint measures were generally similar by treatment arm. Follow-up data by arm for both the BPI and FACT-P TOI endpoints exceeded 78% at Week 10, 66% at Week 19, 58% at week 28, and 50% at week 37.

### Descriptive results

**Fig. 2:**
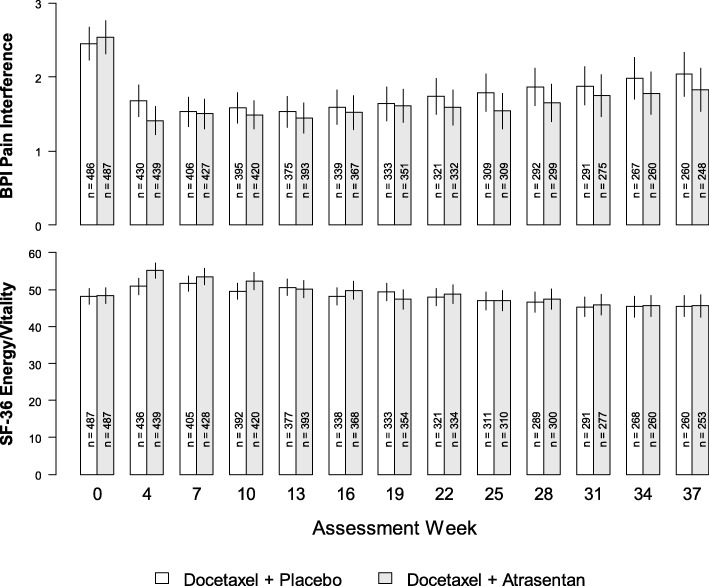
Observed scores by arm over time for the BPI Pain Interference and SF-36 Energy/Vitality scales. Arm and assessment specific sample sizes are shown in the bars. Ninety-five percent confidence intervals for the observed rates are indicated by the vertical lines at the tops of the bars

**Fig. 3:**
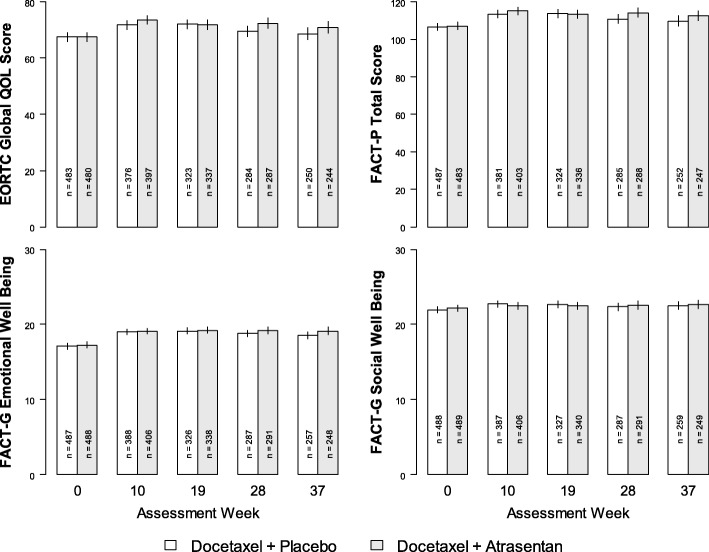
Observed scores by arm over time for Global QOL, FACT-P Total Score, and FACT-G Emotional and Social Well-Being. Arm and assessment specific sample sizes are shown in the bars. Ninety-five percent confidence intervals for the observed rates are indicated by the vertical lines at the tops of the bars

Baselines characteristics were similar for the two treatment arms (Table 1). Mean scores over time for the BPI pain interference score and the SF-36 Energy/Vitality scale by treatment arm are shown graphically in Fig. 2, indicating no strong evidence of treatment arm differences. Similar patterns of limited differences by arm were evident for the EORTC global QOL score, the FACT-P total score, and the social and emotional subscales of the FACT-G (Fig. 3).

**Table 1:** Baseline Patient Characteristics and Baseline Health Related Quality-of-Life^a^

		Docetaxel + Placebo (*n* = 489)			Docetaxel + Astrasentan (*n* = 489)		
Patient Characteristic	Categories	N (%)			N (%)		
Age: median (range)		69.6 (43.2–89.1)			69.4 (40.8–92.0)		
Hispanic ethnicity	Yes	20 (4.1%)			20 (4.1%)		
Race	White	397 (83.6%)			399 (83.3%)		
	Black	64 (13.5%)			72 (15.0%)		
	Asian	12 (2.5%)			6 (1.3%)		
	Pacific Islander	2 (0.4%)			3 (0.6%)		
	Native	1 (0.2%)			1 (0.2%)		
	Unknown	14			10		
Performance status 2 or 3	Yes	39 (8.0%)			35 (7.2%)		
Baseline progression type	Measurable or evaluable	390 (79.8%)			402 (82.2%)		
Extraskeletal metastases	Yes	272 (55.6%)			281 (57.5%)		
Prior Prostatectomy	Yes	142 (29.0%)			165 (33.7%)		
Gleason score	5–6	48 (10.3%)			52 (11.1%)		
	7	133 (28.7%)			138 (29.5%)		
	8–10	272 (58.6%)			271 (57.9%)		
	Missing	25			21		
Pain Medication (worst)	0 – None	14 (4.4%)			18 (5.6%)		
	1 – Non-narcotic	116 (36.7%)			118 (36.5%)		
	2 – Weak opioid	97 (30.7%)			90 (27.9%)		
	3 – Strong opioid	89 (28.2%)			97 (30.0%)		
	Missing	173			166		
Health Related QOL	Categories	N	Mean	SD	N	Mean	SD
Brief Pain Inventory	Worst pain	488	3.4	2.8	489	3.5	3.0
	Worst pain, baseline + 4 week average	434	2.8	2.3	440	2.7	2.4
	Pain interference	486	2.4	2.5	487	2.5	2.6
	Pain interference, baseline + 4 week average	428	2.1	2.2	437	1.9	2.0
SF-36^b^	Energy/vitality	487	48.2	23.3	487	48.3	24.1
	Energy/vitality, baseline + 4 week average	435	49.5	20.7	437	51.7	21.0
FACT-P^c^	Trial outcome index	487	67.5	17.4	484	67.5	17.9
	FACT-G total score	487	77.2	15.7	486	77.7	16.5
	FACT-P total score	487	106.5	22.0	483	107.0	23.0
EORTC QLQ-C30^d^	Global quality of life score	483	63.7	22.7	480	63.6	22.6

^a^Percentages shown among those with known date only

^b^Medical Outcomes Study 36 Item Short Form Health Survey

^c^Function Assessment of Cancer Therapy – Prostate Cancer

^d^European Organisation for Research and Treatment of Cancer – Quality of Life Questionnaire

### Clinically significant change

The baseline/4 week average worst pain score was < 2 for 170 patients on the DPP arm and 183 patients on the DPA arm. Baseline or 4 week data were missing for an additional 55 patients on DPP and 49 patients on DPA. Total missing data for the pain palliation endpoint did not differ by arm (*p* = .70). In total, 521 patients (96% of the accrual goal of *n* = 540) had sufficient data to examine pain palliation. Successful palliation of baseline pain was achieved for 41.7% (110/264) of patients on the DPP arm and 44.0% (113/257) on the DPA arm. This difference by arm of 2.3% did not achieve our pre-specified target of 15% and was not statistically significant (*p* = .66). Determining the “true” number of patients meeting clinically significant change in worst pain is difficult due to the multifaceted definition of change in pain and the missing data in these various components (e.g., the Pain Medication Log). However, including criteria for clinically significant change is critical for more accurate interpretation of these results.

Many fewer patients had missing data for the examination of clinically meaningful change in functional status since all patients had adequate baseline scores to potentially achieve a clinically meaningful difference in functional status, and only a single baseline and follow-up assessment were required. The proportion of patients experiencing a clinically meaningful change in functional status between baseline and follow-up was 24.2% (118/487) on the DPP arm and 28.7% (139/484) on the DPA arm; this difference was not statistically significant (*p* = 0.13) and did not meet our 6-point hypothesized treatment arm difference for functional status.

### Longitudinal analysis models

**Fig. 4:**
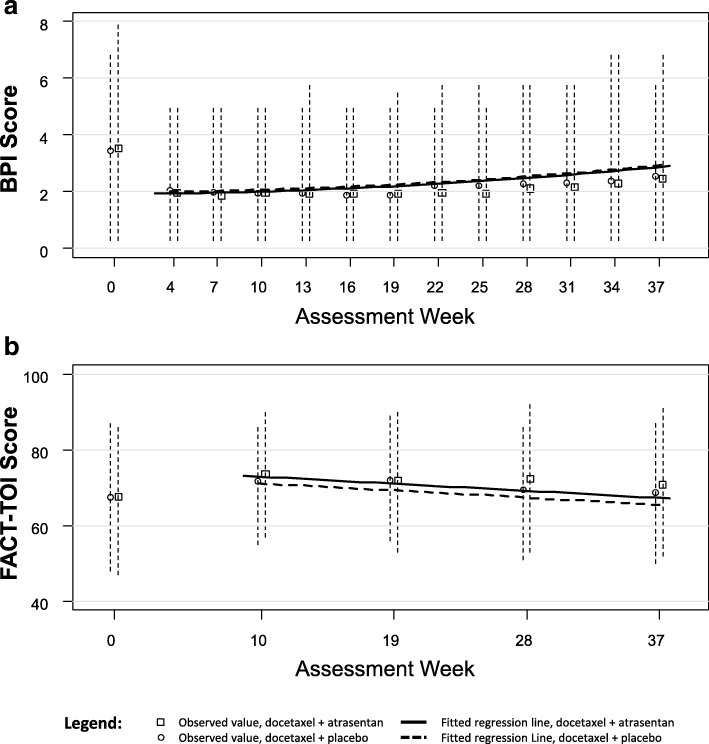
Mean observed BPI Worst Pain and FACT-P TOI scores over time. The fitted lines from the linear mixed models regressions are also shown. The dashed vertical lines show the spread of the observed data by arm and assessment time using the 75% intraquartile range

Patterns of BPI worst pain and the FACT-P TOI score by arm over time are illustrated in Fig. 4. Observed means over time indicated limited evidence of differences by arm. We identified the best models as described above. For neither endpoint was there evidence of different patterns by arm over time (i.e. no interactions; Additional file 1: Table S1). For BPI worst pain, the best model included a quadratic relationship between BPI worst pain scores and time, but there was no difference by arm (*p* = .39; Table 2). For the FACT-P TOI, the best model included a linear relationship between FACT-P TOI scores and time; also, there was evidence that scores differed over time by arm (*p* = .02), although the magnitude of this difference was small (1.78 points on average), suggesting a statistically significant but not clinically meaningful difference.

**Table 2:** Best Model Results for BPI Worst Pain and FACT-P Trial Outcome Index

	Results for Best Model: Row 1 = Model coefficient values Row 2 = *p*-value				
Domain	Intercept	Treatment	Time	Baseline Score	Time-squared
BPI Worst Pain	0.52 (< 0.0001)	−0.10 (0.39)	0.002 (0.78)	0.42 (< 0.0001)	0.0006 (0.001)
FACT-P Trial Outcome Index	32.86 (< 0.0001)	1.78 (0.02)	−0.21 (< 0.0001)	0.60 (< 0.0001)	N/A

Table shows the best model results for the BPI worst pain scores and FACT-P trial outcome index. We used linear mixed models with random effects for the slope and intercept, with a specified compound symmetric correlation structure to account for autocorrelation of serial measures within patients. For each outcome, we examined whether scores differed over time by arm using a treatment by time interaction (both linear and quadratic); if no interaction as present, we modeled time as both linear and quadratic. Best model fit was determined as shown in Additional file 1: Table S1

Cohort plots suggested strong evidence that patients who dropped out earlier reported both more pain and worse function status (Additional file 1: Figs. S1 and S2). The patterns were especially pronounced when patients had missing data after 28 weeks, and thus the indicator variable used in pattern mixture models categorized patients by their missingness patterns at this timepoint (i.e. missing weeks 28–37 vs. not missing weeks 28–37). There was limited or no evidence that patterns of missingness differed by arm for either endpoint. As such, the use of pattern mixture models generate very similar results for treatment arm differences for the BPI Worst Pain score (*p* = .19) and the FACT-P TOI score (*p* = .01).

## Discussion

This study examined PRO/HRQL data for a Phase III clinical trial for patients with mCRPC. Both treatment arms contained docetaxel, the standard of care for patients with mCRPC at the time the study was designed. This trial evaluated the addition of a bone-targeted agent, atrasentan. Given that bone pain is a key aspect in this patient population, even in the absence of improved survival, evaluating pain and other PRO measures in a well-designed Phase III trial with a national sampling of the mCRPC population is critical in order to understand the full results and the ability of the agents to improve/exacerbate symptom status and general functioning.

SWOG S0421 did not identify treatment arm differences in overall survival or progression-free survival [7]. The design of the HRQL analyses included criteria for clinically significant change, which is critical for accurate interpretation. Moreover, the HRQL analyses controlled for measurement error for the worst pain outcomes and evaluated potential impacts of informative missing data in sensitivity analyses. In summary, the DPA arm did not demonstrate the hypothesized change in clinically meaningful pain palliation or functional status improvement compared to the DPP arm. The longitudinal analyses showed little or no difference in worst pain scores over time, but a statistically significant yet small difference in functional status. Additional secondary endpoints also showed little difference in scores over time by arm. Taken together, the results indicated little evidence that DPA was superior to DPP with respect to HRQL.

The level of trial- and PRO-related detail provided in this manuscript is consistent with PRO reporting standards for randomized trials and outlined by Calvert et al. [10] and addresses concerns raised by Fallowfield et al. [30] regarding insufficient presentation of detail regarding improvements and deterioration in symptom status. Calvert et al. developed the PRO Extension for the CONSORT (Consolidated Standards of Reporting Trials) guidelines used for reporting clinical trial results to provide similar guidelines for the key PRO information that should be routinely reported for a trial with PROs [10]. The intent of this effort was to allow for a more comprehensive interpretation of overall trial results for the full set of stakeholders interested in or affected by trial results. Although the PRO measures and design for this trial were selected before these papers were published, they are generally consistent with these guidelines.

### Limitations

One limitation of SWOG S0421 is the absence of information on the screened (non-enrolled) population. Such a limitation is common among network groups trials given limited resources available to sites. Further, missing data for PROs has been recognized as a problem in trials involving patients with advanced stage disease [29]. Even the best quality control procedures used in a clinical trial cannot prevent missing PRO data, especially for patients with advanced stage disease. Cohort plots and accompanying pattern mixture models were used to more fully address concerns about missing data over time as sensitivity analyses [29]. Although there was strong evidence that patients with missing data had worse HRQL in general, patterns were similar by arm, and thus these sensitivity analyses generated largely similar outcomes as the main linear mixed model analysis. This trial also preceded the introduction of patient-reported adverse event reporting developed by the NCI (the PRO Common Toxicity Criteria for Adverse Events), and thus direct patient-reported input on the experience of individual adverse events beyond pain were not available.[18] Finally, although our accrual goal was achieved for the analysis of pain palliation, many patients were not evaluated due to the absence of pain at baseline or inadequate follow-up data to assess pain palliation using multiple metrics (both BPI worst pain and analgesic use). Nonetheless, the use of serial measures to determine clinically meaningful change in pain palliation is critical for accurate interpretation.

## Conclusions

The PRO measures included in SWOG S0421 did not identify substantial treatment arm differences for pain and functional status. However, with any new study, we do not know the extent to which patients will report improved or deteriorated status. Therefore, we encourage researchers to include as comprehensive a PRO assessment as resources will allow. The PRO data can be of use to a wider group of “consumers” of the clinical trial’s results. In our case, the treatment arms had similar average effects on patient HRQL.

## Additional file

Additional file 1: **Table S1.** Identifying Best Fit Model for Longitudinal Analyses of BPI Worst Pain and FACT-P TOI Scores, **Figure S1.** Cohort plot of average BPI worst pain scores by missing data patterns and arm, **Figure S2.** Cohort plot of average FACT-P TOI scores by missing data patterns and arm. (DOCX 115 kb)
