# Supplementary material for: Patient-reported outcomes for patients with metastatic castration-resistant prostate cancer receiving docetaxel and Atrasentan versus docetaxel and placebo in a randomized phase III clinical trial (SWOG S0421)
Source: J Patient Rep Outcomes. 2018 Jun 13;2:27. doi: 10.1186/s41687-018-0054-5 (PMC5997724; doi:10.1186/s41687-018-0054-5)

**Supplementary Material**

Patient-reported Outcomes for Patients with Metastatic Castration-Resistant Prostate Cancer Receiving Docetaxel and Atrasentan versus Docetaxel and Placebo in a Randomized Phase III Clinical Trial (XXXXXX)

**Table S1:** Identifying Best Fit Model for Longitudinal Analyses of BPI Worst Pain and FACT-P TOI Scores

|  | Row 1 = Fit statistic (-2 Log Likelihood), model degrees of freedom  Row 2 = Difference in fit statistic compared to specified nested model, difference in degrees of freedom, p-value by chi-square  Row 3 = Difference in fit statistic compared to alternative specified nested model, difference in degrees of freedom, p-value by chi-square | | | | |
| --- | --- | --- | --- | --- | --- |
|  | Function for Time in Model^#^ | | | |  |
| Domain | Linear,  No Interaction | Linear,  Interaction with Treatment | Time Squared,  No Interaction | Time Squared,  Interaction with Treatment | Best Model |
| BPI Worst Pain | 32853.93, 3 | 32853.57, 4  Vs linear time: 0.36, 1, p=0.55 | 32843.29, 4  Vs linear time: 10.64, 1, p=0.001 | 32842.96, 6  Vs time squared: 0.33, 2, p=.85  Vs linear, no interaction: 10.97, 3, p=.01* | Time Squared, No Interaction |
| FACT-P Trial Outcome Index | 18987.72, 3 | 18984.82, 4  Vs linear time: 2.90, 1, p=.09 | 18986.99, 4  Vs linear time: 0.7, 1, p=.40 | 18981.14, 6  Vs time square: 5.6, 2, p=.054  Vs linear, no interaction: 6.58, 3, p=.09 | Linear, No Interaction |

# Model parameterizations:

Linear, no interaction = intercept, baseline score, treatment, time

Linear, interaction = intercept, baseline score, treatment, time, treatment*time

Square, no interaction = intercept, baseline score, treatment, time, time*time

Square, interaction = intercept, baseline score, treatment, time, treatment*time, time*time, treatment*time*time

* Although the p-value for the comparison with the linear time model is statistically significant, the absence of statistical significance compared to the time squared model suggests that the square function for time dominates the difference, rather than the interaction term, and indicates that the best model fit is the time squared, no interaction model.

**Figure S1:** Cohort plot of average BPI worst pain scores by missing data patterns and arm


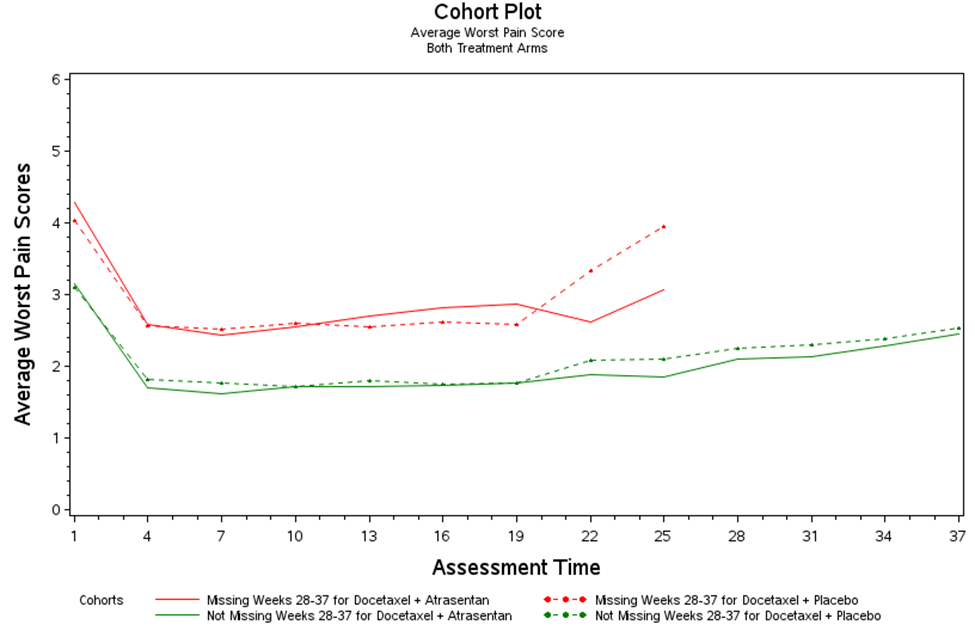


**Figure S2:** Cohort plot of average FACT-P TOI scores by missing data patterns and arm


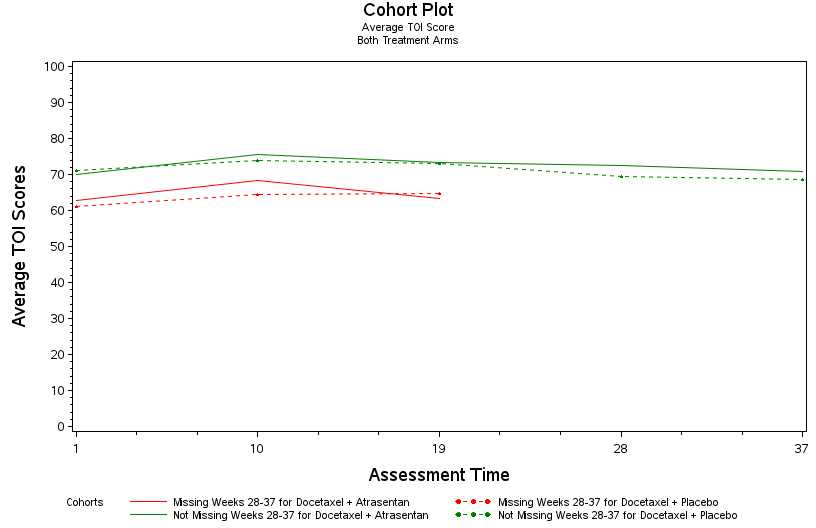

Supplement: Supplementary file 1 — Table S1. Identifying Best Fit Model for Longitudinal Analyses of BPI Worst Pain and FACT-P TOI Scores, Figure S1. Cohort plot of average BPI worst pain scores by missing data patterns and arm, Figure S2. Cohort plot of average FACT-P TOI scores by missing data patterns and arm. (DOCX 115 kb) [file 41687_2018_54_MOESM1_ESM.docx]
